# Reasons for continuing use of Complementary and Alternative Medicine (CAM) in students: a consumer commitment model

**DOI:** 10.1186/s12906-016-1059-3

**Published:** 2016-02-24

**Authors:** Fuschia M. Sirois, Anita Salamonsen, Agnete E. Kristoffersen

**Affiliations:** Department of Psychology, University of Sheffield, Sheffield, UK; Department of Psychology, University of Windsor, Windsor, Canada; National Research Center in Complementary and Alternative Medicine, Department of Community Medicine, UiT the Arctic University of Norway, Tromsø, Norway

## Abstract

**Background:**

Research on continued CAM use has been largely atheoretical and has not considered the broader range of psychological and behavioral factors that may be involved. The purpose of this study was to test a new conceptual model of commitment to CAM use that implicates utilitarian (trust in CAM) and symbolic (perceived fit with CAM) in psychological and behavioral dimensions of CAM commitment.

**Methods:**

A student sample of CAM consumers, (*N* = 159) completed a survey about their CAM use, CAM-related values, intentions for future CAM use, CAM word-of-mouth behavior, and perceptions of being an ongoing CAM consumer.

**Results:**

Analysis revealed that the utilitarian, symbolic, and CAM commitment variables were significantly related, with *r*’s ranging from .54 to .73. A series hierarchical regression analyses controlling for relevant demographic variables found that the utilitarian and symbolic values uniquely accounted for significant and substantial proportion of the variance in each of the three CAM commitment indicators (*R*^*2*^ from .37 to .57).

**Conclusions:**

The findings provide preliminary support for the new model that posits that CAM commitment is a multi-dimensional psychological state with behavioral indicators. Further research with large-scale samples and longitudinal designs is warranted to understand the potential value of the new model.

## Background

Interest in and use of complementary and alternative medicine (CAM) has generally continued to grow over the past several decades, as has our understanding of why people use CAM and their patterns of CAM use. CAM includes a broad and diverse set of healing therapies of differing modalities, practices, and health systems [1] that can be delivered by a trained practitioner or administered as self-care. Much of the early research focused largely on the demographic and health belief factors associated with CAM use, and on distinguishing CAM users from non-users via comparative research. Researchers have however highlighted the need to take a more sophisticated view of CAM use by examining why some individuals might continue or discontinue their use of CAM. Indeed, many individuals who initially try CAM may continue their use, integrating CAM into their health care repertoire. Cross-sectional and qualitative research to date indicates that motivations for continued CAM use differ from those for initial or trial use [2–4], prompting researchers to suggest that these motivations should be separated to better understand why people use CAM [3, 5]. Considering the complexities of factors that might contribute to ongoing use of CAM is therefore important for providing a more comprehensive view of the patterns of CAM use, and for informing both practice and policy.

The question of what keeps people using CAM over time and becoming committed to CAM as a health-care choice has not been adequately addressed by previous research. There are cross-sectional studies that hint at the possible reasons for continued use [3, 6], but they have not articulated what is meant by commitment to CAM, nor have they tested theoretical models derived from extant research. The purpose of the current research was to address this important gap in the research on CAM use by proposing and testing a new model that highlights the factors that contribute to the development of commitment to CAM.

### A new model of commitment to CAM

The concept of commitment is in many ways unique to CAM as it is rarely used in reference to conventional health care. As a dominant form of health care, conventional care is often the default form of health care for many people. In contrast, CAM care involves making a conscious choice to seek care in addition to, but also sometimes in place of, conventional care, and with added costs. In this respect, the repeated and continued use of CAM can be viewed as reflective of this ongoing conscious choice or *commitment* to use CAM as a health-care choice across different health-care situations. Researchers have previously conceptualized CAM commitment primarily in behavioral terms by suggesting that the extent and degree of CAM use [7, 8], or the years of experience with CAM [2, 3] reflect commitment to CAM. However, because CAM use may also be driven primarily by need [3, 9], using the extent and degree of CAM use as a marker for commitment to CAM is likely to be confounded by the presence or absence of health issues that require treatment. What is missing from these conceptualizations of CAM commitment is an acknowledgement of the cognitive and affective dimensions of patients’ experiences with CAM and how CAM use itself may motivate continued use. This is especially important given the known links among beliefs, values, and continuing CAM use [2, 3, 10, 11]. Accordingly, we expand on these views by conceptualizing commitment to CAM as a psychological state with behavioral indicators.

We further suggest that the concept of commitment to CAM may be well understood from the lens of consumer psychology as patients are increasingly being viewed as consumers by the health care industry and policy makers. A consumer psychology approach is especially relevant to CAM because most CAM therapies are not covered by insurance but are paid for out-of-pocket by consumers, and their use is therefore elective. CAM commitment closely resembles a construct from consumer psychology, brand commitment or the degree to which a “consumer is emotionally attached to the relationship with a particular brand in a product class” [12]. From this perspective CAM can be viewed as a particular “brand” of health care that the consumer chooses.

Our new model of CAM commitment is based on a model of brand commitment developed, tested and validated across three independent samples of consumers by Wang [12]. According to this model, commitment to a particular brand develops from two types of positive experiences with the brand: 1) a functional route associated with utilitarian needs and motivations that are reflected through positive outcomes, brand satisfaction and trust, and 2) a symbolic route that involves a perceived “fit” between the consumer’s values and the brand (value congruency). Together these two routes lead to commitment, which is evidenced by intentions to purchase and subsequent repeat purchases, recommendations of the “brand” to others or word-of-mouth (WOM) behavior, and a sense of belongingness to the community of other brand users. Empirical work supports the suggested relations in this model. For example, a survey of over 1,000 consumers found that value congruency was predictive of service-brand commitment [13], and WOM behavior has been found to be a robust indicator of brand commitment [14, 15]. With respect to CAM use, WOM behavior has mainly been examined as a source of CAM information that people draw upon when deciding whether or not to use a particular CAM modality. For example, a number of studies suggest that social relationships are a valued and common source of information in the decision to use CAM [2, 16, 17]. Bringing together this evidence with theory and research on brand commitment, we propose that those who serve as a source of CAM information for family and friends interested in trying CAM do so because they are committed to using CAM as form of health-care and are therefore happy to share their CAM experiences and knowledge.

**Fig. 1:**
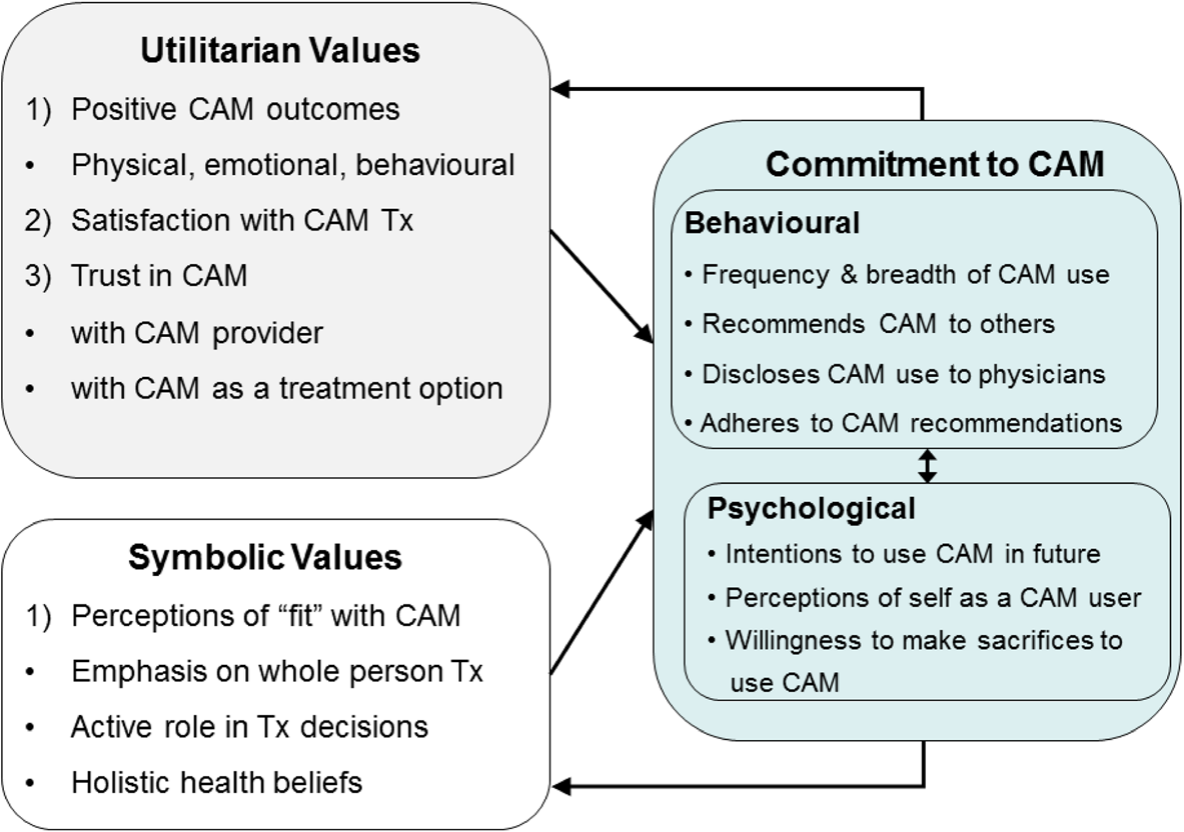
CAM Consumer Commitment Model

Figure 1 outlines the new model of CAM commitment and the utilitarian and symbolic values that are involved. The utilitarian and symbolic values proposed to underlie the route to CAM commitment are consistent with Lupton’s [18] proposition that health care decisions are not simply a rational response to perceived need, and that health care has not only “use” or practical value but also abstract or symbolic value. Empirical evidence supports the notion that utilitarian values are important precursors of CAM commitment. For example, trust in the provider is linked to continued CAM use [4, 19], and trust in CAM treatments as a treatment option motivates use [20]. Other positive physical, emotional, and behavioral outcomes from CAM treatment may play a role in commitment [4], even if such outcomes are not anticipated. When expected, they may reinforce patients’ decision to use CAM and enhance satisfaction [4]. With respect to symbolic values, a perceived congruency or “fit” between an individual’s belief system and that of CAM can be an important motivator of CAM use [2, 3, 5, 10]. Support for this idea also comes from a systematic review of beliefs associated with CAM use, which found that beliefs about control and participation, a holistic view of health, and a desire for natural, non-invasive treatments, predict CAM use after controlling for demographic and clinical variables [21]. In the new model CAM commitment is demonstrated through both behavioral and psychological components, which are linked. The behavioral component of CAM commitment includes the breadth and frequency of CAM use as suggested by other researchers [7, 8], disclosure of CAM to other health care professionals as noted in previous research with this model [22], adherence to CAM recommendations, and CAM WOM behavior, a concept that has not been previously examined with respect to continued use of CAM. Because behavioral intentions are a known predictor of future behavior [23], the psychological component includes intentions for future CAM use as well as a willingness to spend out of pocket for CAM, and perceiving oneself as a CAM user.

A preliminary test of portions of this model focusing on one behavioral component of CAM commitment, CAM disclosure, provides some support for the proposed role of utilitarian and symbolic values in commitment to CAM. Across student and community adult general medical samples, specific utilitarian and symbolic values were significantly associated with disclosure of CAM use to conventional medicine general practitioners and specialists [22]. Though promising, this previous work examined only one behavioral component of CAM commitment suggested by the CAM Consumer Commitment model, and select values rather than a more comprehensive range of perceptions of fit with CAM.

### The current research

Although previous research indicates that the symbolic and utilitarian values identified in the new CAM commitment model may each be linked to CAM use separately, they have not been tested collectively or as part of a more comprehensive theoretical framework specifically examining different dimensions of CAM commitment. The aim of the present research was therefore to provide a more complete test of the new model of CAM consumer commitment. Specifically, we posited that select utilitarian and symbolic values would be associated with a set of behavioral and psychological markers of commitment to CAM.

We chose a select number of variables from the CAM consumer model that had not been extensively tested with respect to continued CAM use. Previous work on continued CAM use has tested and found support for two of the behavioral markers of commitment to CAM suggested by the CAM consumer commitment model, frequency and breadth of CAM use [3, 8], and disclosure of CAM use [22]. Word of mouth behavior (WOM) was chosen as the behavioral marker of CAM commitment as it has received little or no attention with respect to continued CAM use in previous research. We chose identifying oneself as a CAM user, and intentions to use CAM in the future as the psychological markers of CAM commitment to examine. To the best of our knowledge neither variable has been investigated with respect to continued CAM use. As discussed previously, there are strong theoretical reasons for expecting each to reflect the psychological commitment to CAM. We chose trust in the effectiveness of CAM as the utilitarian value to examine as it was decided to be the best marker of this model component. For example, trusting CAM implies having had previous positive experiences and satisfaction with CAM, two indicators of CAM commitment dimensions from the model that have been associated with long-term CAM use in previous research [22]. For the symbolic values we chose four perceptions of “fit” with CAM linked to CAM use by previous research: emphasis on whole person treatment identified by previous research: having an active role in treatment decisions, valuing being treated as an equal partner in health-care decisions, and holistic/natural health beliefs [3].

## Method

### Participants and procedure

Following clearance from the University of Windsor Research Ethics Board, a sample of undergraduate students was recruited to participate in a study on health behaviors and beliefs. All procedures followed were in accordance with the ethical standards of the responsible committee on human experimentation (institutional and national) and with the Helsinki Declaration of 1975, as revised in 2000. Informed consent was obtained from all participants included in the study. Participants were recruited in the Fall of 2008 from the University of Windsor, a mid-sized university in Southwestern Ontario, Canada via notices placed on a university participant pool web page. The study notices provided a link to a dedicated web page that directed participants to the online survey housed on a secure university server. Participants indicated their consent to participate in the study by clicking an “I agree” button on the online consent form and were given course research credit for their participation. The study notice was posted for the Fall semester with the aim to recruit as many participants as possible from the participant pool during this time frame.

### Measures

Participants completed an online survey that included questions about demographic information and their CAM use in the previous 6 months, and measures of utilitarian and symbolic values for CAM commitment, as well as three indicators of CAM commitment suggested by the new model.

#### CAM use

Participants were asked if they were currently using provider-based CAM, and if so they reported whether they had ever visited any of the CAM providers listed. CAM providers included commonly used modalities from the manipulative and body based practices (chiropractic, massage therapy, reflexology), energy medicine (acupuncture, reiki), and whole medical systems (homeopathy, naturopathy, Traditional Chinese medicine, Ayurvedic Medicine), as classified by NCCIH [24] and the Cochrane group CAM classification [25]. Three spaces were also provided for participants to check and list any other provider-based CAM they had used, with examples given (aroma therapy, biofeedback, yoga, etc.). They were also asked if they had ever used any other CAM on their own, i.e., without the assistance of a health-care provider, with the examples of yoga, meditation, herbal remedies or other natural health products given. To obtain a more complete profile of the participants’ CAM use, participants also reported whether they used CAM regularly, occasionally, rarely or not at all. This CAM use checklist has been used previously in other CAM research [22].

#### Utilitarian values

Trust in CAM was assessed with the single item “I trust that complementary/alternative medicine will be effective for my needs.” Participants rated their agreement with this statement on a 6-point Likert-type scale ranging from 1 (*Strongly disagree*) to 6 (*Strongly agree*). This item has been used in previous research to assess the beliefs associated with CAM use [2, 6].

#### Symbolic values

The different dimensions of symbolic values for CAM use were assessed with four items from previous research on motivations for CAM use, which have demonstrated good concurrent validity with other meaning related CAM motivations, and good predictive validity of the degree of CAM use [2, 3, 6]. The statements included “I believe that complementary/alternative medicine allows me to take a more active role in maintaining my health”, “I value the emphasis that complementary/alternative medicine places on treating the whole person”, “I value the way that complementary/alternative medicine practitioners treat me as an equal partner in managing my health”, and “I prefer taking a natural approach to health and healing”. Participants were asked to rate their agreement with each statement on a 6-point Likert-type scale ranging from 1 (*Strongly disagree*) to 6 (*Strongly agree*), specifically with respect to their reasons for using CAM. An overall symbolic CAM values scale was created from these items by taking the mean score of the 4 items. The internal consistency was good, with a Cronbach alpha of .84.

#### Commitment to CAM

Two different aspects of the psychological component of commitment to CAM were assessed. Intentions to use any form of CAM in the future were assessed on a 9-point scale ranging from 1(*Not at all likely to use again*) to 9 (*Extremely likely to use again*). Perceptions of being an ongoing CAM consumer were assessed with the single item “Using complementary/alternative medicine is part of my lifestyle” that was rated on a 6-point Likert-type scale ranging from 1 (Strongly disagree) to 6 (Strongly agree). Both items were created for the current study to assess aspects of CAM commitment as suggested by the CAM consumer model.

The behavioral component of commitment to CAM was assessed with two items focused on both past and future word of mouth (WOM) behavior created for the current study and informed by past research on CAM decision information sources [2, 16]. Participants were asked to respond to the question “How often in the past 3 months have you recommended complementary/alternative medicine to other people you know (e.g., friends, family, co-workers)?” on a 4-point scale ranging from 1 (never) to 4 (very often). They also rated the question “How likely are you to recommend complementary/alternative medicine to other people (e.g., friends, family, co-workers)?” on a 9-point scale ranging from 1 (Not at all likely to recommend) to 9 (Extremely likely to recommend). Because the 2 items were on different rating scales, the scores for the 2 items were first standardized by taking a z-score, and then averaged to create a WOM index. The two standardized scores for each item were significantly correlated, *r* = .62, *p* < .000.

### Data analyses

Data were first screened and duplicates and surveys that were missing 20 % or more of the required responses were excluded from the analyses. Respondents were classified as non-CAM users or CAM users, based on 1) whether they considered themselves current CAM users, and 2) their use of provider-based CAM in the previous 6 months.

After testing the distribution of the model variables for meeting the assumptions of normality, correlational analyses were conducted among the measures of symbolic and utilitarian values, and the CAM commitment indicators for descriptive purposes. To determine the collective and independent contributions of the utilitarian and symbolic values to the three CAM commitment dimensions, a series of hierarchical multiple regressions were conducted. In all analyses, demographic variables (age, sex) were entered in the first step, and the predictor variables (symbolic and utilitarian values) in the next step, as previous research has demonstrated that CAM users tend to female [26–29], middle-aged [30], and that CAM use for men and women varies as a function of age [31]. Although empirical investigations also indicate that CAM users are highly educated [28, 32, 33], education was not entered as a covariate as the participants all had some level of university education. Significance level was set at *p* < 0.05, and all analyses were conducted with SPSS version 21.

## Results

### Participant characteristics

A total of 359 participants completed the survey. Of these, 44 % (*N* = 159; Mean age = 21.9, SD = 5.02) reported CAM use in the previous 6 months and therefore were selected as the sample analysed for the current research. The majority of the participants were female (88.7 %) employed part-time or not at all, and had at least some university education.

### CAM use

**Fig. 2:**
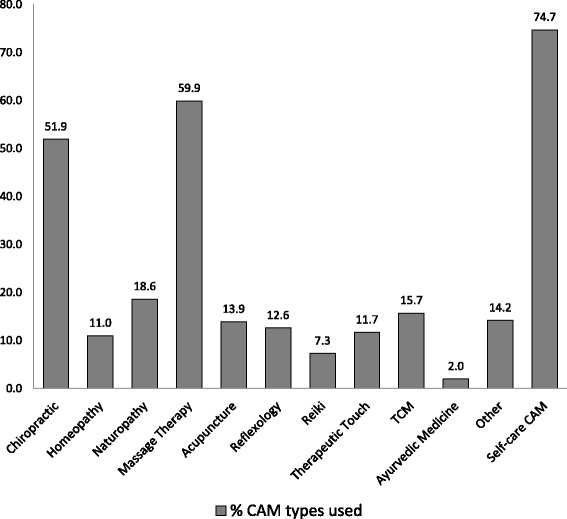
Percentage of participants who used each form of complementary and alternative medicine (CAM)

Participants (40.9 %) indicated that they used CAM occasionally, rarely (39.6 %), or regularly (19.5 %). Figure 2 presents an overview of the types of CAM used in the sample. Although all participants had used some form of provider-based CAM in the previous 6 months, the majority of the sample had also used a self-care CAM, with yoga and herbalism being the most reported types. Massage therapy was the most commonly used provider delivered CAM, followed by chiropractic, naturopathy, Traditional Chinese medicine, other CAMs, and acupuncture.

### Testing the CAM consumer commitment model

All model variables met the assumptions for normality. Table 1 presents the bivariate correlations among the CAM Consumer Commitment Model variables. The utilitarian and symbolic value variables were positively correlated with each other as well as with each of the three CAM commitment dimensions. The three CAM commitment variables were in turn positively correlated with each other. On average, participants reported a moderately high degree of intentions to continue CAM use in the future.

**Table 1:** Bivariate Correlations Among the CAM Consumer Commitment Model Variables (*N* = 159)

Variable	1	2	3	4	5
1. Utilitarian values (trust in CAM)	---				
2. Symbolic values	.73**	---			
3. Continued CAM use intentions	.65**	.61**	---		
4. CAM consumer self-perceptions	.67**	.69**	.64**	---	
5. WOM behavior index	.54**	.55**	.70**	.57**	---
Mean	4.25	4.37	7.10	3.94	0.00
Standard deviation	1.14	1.01	1.81	1.32	0.90

*Note: WOM* word of mouth behavior; **p < .001

The hierarchical regression analyses revealed that the utilitarian values variable (trust in CAM), and symbolic values each explained a unique and substantial proportion of the variance in the three dimensions of CAM commitment (see Table 2), after controlling for the two demographic variables. Together the utilitarian and symbolic values accounted for an additional 44 % of the variance in intentions to continue to use CAM, 53 % of the variance in perceptions of being a CAM consumer, and 35 % of the variance in word of mouth beh24avior. Among the demographic variables entered in to the regression models, only age was significant, but only for the CAM consumer self-perceptions regression model.

**Table 2:** Hierarchical Regression Analyses Testing the Contribution of Utilitarian and Symbolic Values to Dimensions of the CAM Consumer Commitment Model (*N* = 159)

Outcome	Continued CAM use intentions		CAM consumer self-perceptions		WOM behavior index	
Predictor	Step 1 β	Step 2 β	Step 1 β	Step 2 β	Step 1 β	Step 2 β
Age	.16	.11	.18**	.15**	.15	.13
Gender	.05	.02	.02	-.04	.03	-.01
Utilitarian values (trust in CAM)	---	.47**	---	.35**	---	.33**
Symbolic values	---	.25**	---	.44**	---	.31**
	Step 1	Step 2	Step 1	Step 2	Step 1	Step 2
*R* ^2^	.03	.47	.03	.57	.02	.37
*F*	2.06	31.89**	2.53	47.22**	1.69	21.11**
Δ *R* ^2^	.03	.44	.03	.53	.02	.35
Δ *F*	2.06	60.04**	2.53	88.89**	1.69	39.64**

Note: The degrees of freedom (df) for the *F* value vary according to the number of predictors entered in each step: two predictors, first step, df = (2, 287); second step with four predictors, df = (4, 280); ** *p* < .01

## Discussion

The aim of the current study was to provide a comprehensive test of a new model of CAM consumer commitment that builds on principles from consumer psychology. The analyses provided support for this new model by finding that utilitarian and symbolic values were each significantly associated with intentions to use CAM in the future, self-perceptions of being a CAM consumer, and word of mouth (WOM) behavior. Importantly, each of the proposed consumer values explained unique and substantial variance in the three markers of commitment to CAM over the effects of demographic variables.

Whereas previous research has conceptualized and examined commitment to CAM in terms of the frequency and breadth of CAM use [3, 7, 8], this is the first study to view commitment to CAM as a psychological state with behavioral indicators and find supportive evidence for the role of consumer values for explaining why some individuals may continue to use CAM as part of their health-care repertoire. Specifically, we found that trusting in the effectiveness in CAM, and perceiving a fit between one’s own values for health-care and CAM were associated with both psychological and behavioral indicators of commitment to CAM use. Although the correlational analyses revealed that these utilitarian and symbolic values were significantly related to each other, regression analyses supported their unique contribution to CAM commitment by finding that each accounted for significant variance in the markers of CAM commitment. This consistency in the associations with the CAM commitment variables (betas ranging from .25 to .47 across all models) further supports the importance of both of these values for understanding the different dimensions of CAM commitment in the new model. This is also the first study to identify WOM behavior as an important indicator of commitment to CAM, rather than simply as a source of CAM information. A corollary to this is that CAM commitment is multidimensional and may be best captured by assessing both psychological and more indirect behavioral indicators such as WOM behavior, rather than simply by examining CAM usage patterns. This finding is also consistent with sociological understandings of peoples’ commitment to CAM, which highlights the emergence of a new value system that is congruent with the philosophical underpinnings of CAM [11], and that CAM users are consumers of health care who are characterized by individual autonomy, responsibility, and values and acting in rather complex markets of health care [34].

This research makes an important contribution to previous theoretically driven research on the reasons and factors associated with CAM use that has mainly used the socio-behavioral model (SBM) [35] to understand motivations for CAM use [3, 9, 36]. Although the SBM provides a general guide for delineating the sequence of factors (predisposing, enabling, and need) that may result in a decision to use CAM, it does not sufficiently account for factors involved in the development of enduring motivations to use CAM. Moreover, the focus of the SBM is understanding utilization of health care rather than the development of commitment, a psychological state proposed to motivate continued use of CAM. The new CAM Consumer Commitment model therefore takes CAM use research into a new area by providing researchers and practitioners with a conceptual framework for understanding why people continue to use CAM, and suggesting the routes through which a state of commitment to CAM use may develop.

### Limitations and strengths

Although novel, the findings from the current study should be considered in the context of several limitations. The cross-sectional nature of the study precludes making any conclusions about causality, and therefore replication using more sophisticated methodology is necessary to confirm the proposed relationships among the new CAM commitment model variables suggested by the current findings. Longitudinal work following a group of new CAM consumers over time would provide a more rigorous test of the findings suggested by the current study, as well as address the issue of reciprocal causality between continued use and CAM-related values suggested by the model. However, qualitative work examining the motives of committed and non-committed CAM consumers would also provide further validation of the proposed relationships among health-care beliefs and CAM commitment from the new model. The findings will also need to be tested with other samples, as the student sample may not be representative of other CAM consumer samples. Although other research has demonstrated that the factors associated with CAM use among students tend to mirror those from more representative samples [22], the out-of-pocket costs of most CAM therapies and practitioners may make CAM less affordable for this population. Nonetheless, the previous test using the CAM commitment model as a guiding conceptual lens in both student and community adult samples found that the utilitarian and symbolic factors associated with a behavior related to CAM commitment (i.e., CAM disclosure) in the student sample, were comparable to those in the community adult sample [22], suggesting that potential financial barriers associated with CAM use may not necessarily influence the reasons for continued CAM use suggested by the CAM commitment model. Although the study sample had used provider-based CAM in the previous 6 months, self-care CAM such as yoga was also used by a majority of the sample in addition to provider-based CAM, with more mainstream CAMs as the most commonly used provider-based CAMs. Replication of these findings with larger and more diverse samples of CAM consumers is necessary to more fully evaluate their generalizability and to assess the possible boundary conditions of the new model of commitment to CAM.

Future research with the CAM consumer commitment model would also benefit from taking a sophisticated statistical approach, such as structural equation modelling, to provide a more comprehensive test of the interrelations between the latent constructs suggested by the model. Using this approach would also permit a simultaneous test of all the model variables, which collectively could account for greater variance in commitment to CAM than what was found in the current study.

Despite these limitations, the current study has a number of strengths worth noting. The student sample was recruited through a formal and secure participant pool, which increases the generalizability to other similar student populations that traditionally participate for bonus points. Including a variety of both provider-based and self-care CAM consumers also increases the generalizability of the findings across different CAM modalities. It could be argued that the shift towards integrating CAM therapies into conventional care in recent years could influence the unique branding of CAM and thus the relevance of this model. However, the theoretical backgrounds and philosophies of healing that characterize many CAM modalities are distinct from those of conventional medical care and thus justify a branding of CAM as a unique health-care option even when offered within the context of conventional care. Thus, the introduction and testing of a new conceptual model of consumer commitment to CAM use makes an important contribution to the research on CAM use patterns by providing a framework to guide future research on the values that may contribute to the development of commitment to CAM use over time.

From the perspective of policy and practice, this new framework has the potential to explain current health-care decisions and predict people’s future decision-making. The Consumer Commitment to CAM model provides more systematic and specific knowledge on CAM related health behavior and decision-making that can be applied to different groups of healthcare users than current available models. Such knowledge may be useful information both in practitioner-patient communication in clinical encounters to increase understanding of who may be more or less likely to adhere to CAM treatment recommendations, and to policy-makers in their development of future patient-centered, seamless and targeted public health care programs [37, 38].

## Conclusions

By testing a new model of Consumer Commitment to CAM as a psychological state with behavioral indicators, this study contributes new knowledge to the research on CAM use patterns relevant to researchers and health policy makers in complex health societies. Trusting in the effectiveness in CAM, and perceiving a fit between one’s own values for health-care and CAM seems to be associated with both psychological and behavioral indicators of commitment to CAM use. This may have important implications for other aspects of commitment to CAM including CAM adherence and willingness to pay out-of-pocket for CAM services and products. The CAM Consumer Commitment Model, therefore, warrants further validation and testing in large-scale studies to better understand its potential application and value for policy and practice.

## Abbreviations

CAM: Complementary and alternative medicine
WOM: Word-of-mouth
NCCIH: National Center for Complementary and Integrative Health
SBM: Socio-behavioral model
